# Crystal structure of *N*-{*N*-[*N*-(*tert*-but­oxy­carbon­yl)-l-α-aspart­yl]-l-α-aspart­yl}-l-α-aspartic acid 1^4^,2^4^,3^4^-trimethyl ester 3^1^-2-oxo-2-phenyl­ethyl ester {Boc-[Asp(OMe)]_3_-OPac}

**DOI:** 10.1107/S2056989019004596

**Published:** 2019-04-09

**Authors:** Takuma Kato, Saki Kishimoto, Akiko Asano, Mitsunobu Doi

**Affiliations:** a Osaka University of Pharmaceutical Sciences, 4-20-1 Nasahara, Takatsuki, Osaka 569-1094, Japan

**Keywords:** crystal structure, peptide, hydrogen bonding, homopeptide

## Abstract

In the title homotripeptide {Boc-[Asp(OMe)]_3_-OPac}, C_28_H_37_N_3_O_13_, all peptide bonds adopt an *s-trans* conformation with respect to the N—H and C=O groups. In the crystal, N—H⋯O hydrogen bonds result in an infinite parallel β-sheet structure running along the *b-*axis direction. The Boc protecting group at the *N*-terminus of the peptide is disordered over two sites with occupancy factors of 0.504 (5) and 0.496 (5).

## Abstract 2

In the title homotripeptide of l-aspartic acid β-methyl ester [Asp(OMe)], all peptide bonds adopt an *s-trans* conformation with respect to the N—H and C=O groups. In the crystal, N—H⋯O hydrogen bonds result in an infinite parallel β-sheet structure.

## Chemical context   

In peptide stereochemistry, many studies have been performed in order to control the peptide’s secondary structure. Among them, controlling helix handedness can greatly impact the design of some biological mol­ecules such as mol­ecular switches and the pharmaceutical lead like protein–protein inter­action inhibitors (de Zotti *et al.*, 2014 ▸). Blout & Karlson (1958 ▸) reported that the homopolymer of aspartic acid β-benzyl ester existed as a left-handed helix in solutions of halogenated hydro­carbones (CHCl_3_ and CCl_2_COOH), although early studies have clearly shown that a classical ordered α-helix structure in all-*L* peptides is right handed because of the absolute configuration of their α-amino acid building blocks. Subsequently, this research topic was expanded by many other groups, and numerous β-esters have been investigated (Toniolo *et al.*, 1968 ▸). In this work, we focus on the homo-tripeptide of Asp(OMe) as a simple model of the homo-polypeptide because of the difficulties in collecting X-ray diffraction data for polypeptides.
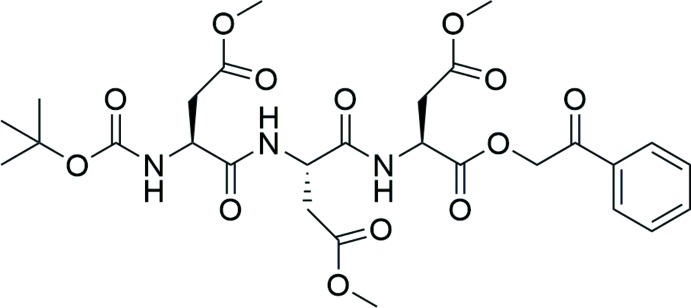


## Structural commentary   

**Figure 1:**
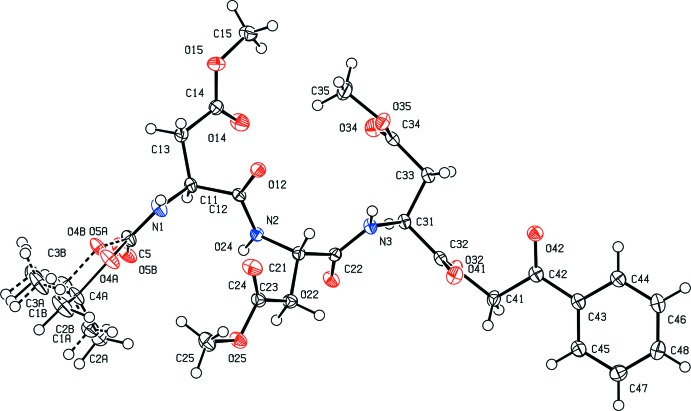
The mol­ecular structure of the title compound with displacement ellipsoids drawn at the 50% probability level. The minor component of the disordered group is drawn with dashed lines.

Table 1 ▸ shows selected torsion angles for the title tripeptide. The mol­ecular structure of the tripeptide adopts an extended conformation of the backbone chain (Fig. 1 ▸) with the φ- and ψ-torsion angles being φ_1_ = −122.49 (17)°, φ_2_ = −116.98 (16)°, φ_3_ = −84.60 (18)°, ψ_1_ = 86.49 (17)° and ψ_2_ = 112.58 (16)°, residing in the β region of the Ramachandran plot. All three consecutive peptide residues are in an *s-trans* conformation with the ω-torsion angles being ω_0A_ = 169.2 (3)°, ω_0B_ = −167.9 (2)°, ω_1_ = −168.62 (12)° and ω_2_ = −173.95 (13)°. The side chains of Asp(OMe), N2—C21—C23—C24 and N3—C31—C33—C34, adopt a *g* ^+^ conformation [χ_2_ = −66.56 (19)° and χ_3_ = −57.72 (17)°], which is the most preferred conformation of aspartic acid (Chakrabarti & Pal, 2001 ▸), while the terminal side chain of Asp(OMe), N1—C11—C13—C14, adopts a *t* conformation [χ_1_ = 172.69 (15)°].

## Supra­molecular features   

**Figure 2:**
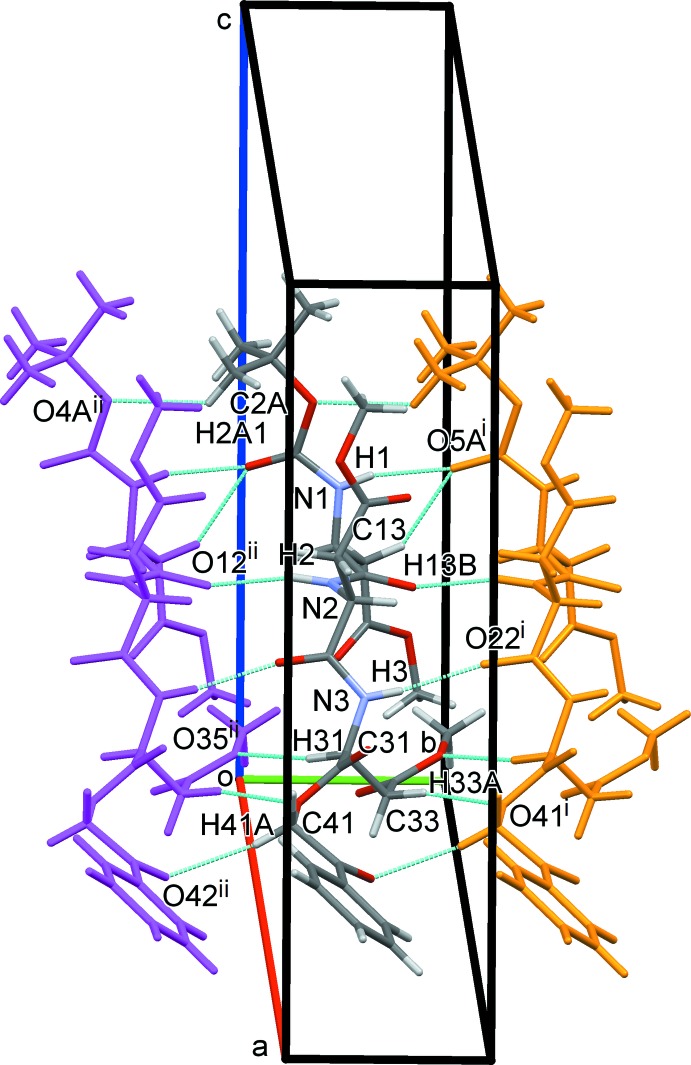
A packing diagram of the title compound, showing the infinite parallel β-sheet structure along the *b-*axis direction formed by the N—H⋯O and C—H⋯O hydrogen bonds (blue dashed lines). Only the major disorder component is shown. [Symmetry codes: (i) *x*, *y* + 1, *z*; (ii) *x*, *y* − 1, *z*.]

**Figure 3:**
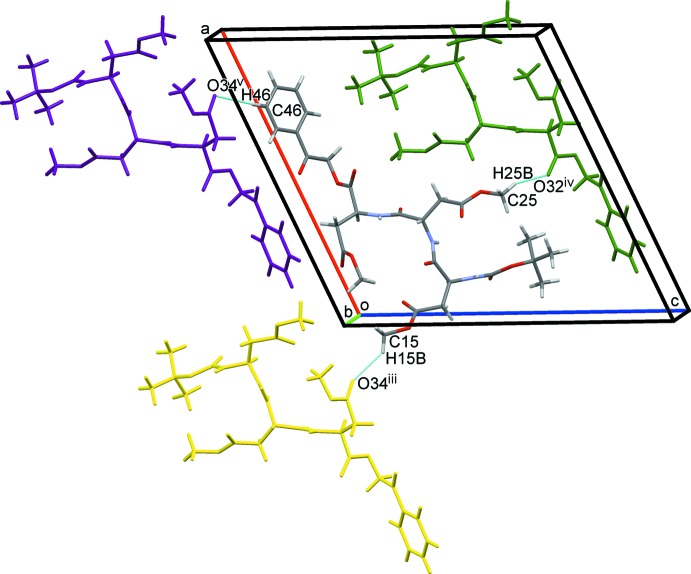
A packing diagram of the title compound viewed approximately along the *b* axis, showing the C—H⋯O hydrogen bonds between the β-sheets (blue dashed lines). Only the major disorder component is shown. [Symmetry codes: (iii) −*x*, *y* + 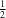, −*z*; (iv) −*x* + 1, *y* − 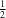, −*z* + 1; (v) −*x* + 1, *y* + 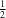, −*z*.]

In the crystal, all of the N atoms in the tripeptide are engaged in inter­molecular N—H⋯O hydrogen bonds [N1—H1⋯O5*A* ^i^, N1—H1⋯O5*B* ^i^, N2—H2⋯O12^ii^ and N3—H3⋯O22^i^; Table 2 ▸]. These hydrogen bonds and some C—H⋯O hydrogen bonds (C2*A*—H2*A*1⋯O4*A* ^ii^, C13—H13⋯O5*A* ^i^, C13—H13⋯O5*B* ^i^, C31—H31⋯O35^ii^, C33—H33*A*⋯O41^i^ and C41—H41*A*⋯O42^ii^; Table 2 ▸) link the mol­ecules, forming an infinite parallel β-sheet structure along the *b-*axis direction (Fig. 2 ▸). Other C—H⋯O hydrogen bonds [C15—H15*B*⋯O34^iii^, C25—H25*B*⋯O32^iv^ and C46–H46⋯O34^v^; Table 2 ▸] further link the β-sheets, forming a three-dimensional network (Fig. 3 ▸).

## Database survey   

A search of the Cambridge Structural Database (Version 5.39, updated November 2017; Groom *et al.*, 2016 ▸) for homo-dipeptides and tripeptides of Asp and Asp β-esters yielded zero hits. A search for dipeptides having an Asp(OMe) residue yielded two hits [DOBWIA (Fuganti *et al.*, 1986 ▸) and GABVEK (Mcharfi *et al.*, 1986 ▸)]. DOBWIA, an α-l-aspartyl-l-phenyl­alanine derivative, has an extended intra- and inter­molecular hydrogen-bonding network. GABVEK, an α-l-prolyl-l-aspartic acid derivative, shows a βI-turn conformation.

## Synthesis and crystallization   

**Figure 4:**
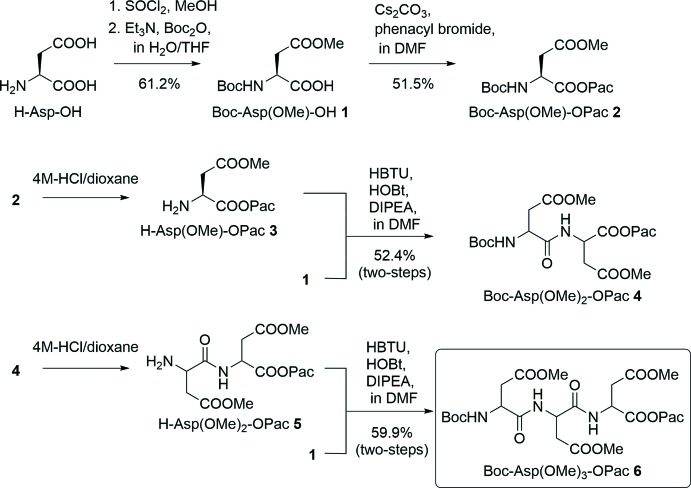
Synthetic scheme for the title Asp(OMe) homo-tripeptide compound, **6**.

The synthesis of the title homotripeptide, **6**, was performed according to the scheme in Fig. 4 ▸.

Compound **1** was synthesized from l-aspartic acid according to a previously described method (Reddy *et al.*, 2011 ▸; Ollivier *et al.*, 2010 ▸). Yield: 61.2% ^1^H NMR (400 MHz, CDCl_3_): δ 1.45 (*s*, 9H, Boc *t*-but­yl), 2.83–2.89 (*m*, 1H, Asp βH), 3.03–3.08 (*m*, 1H, Asp βH), 3.72 (*s*, 3H, Asp OCH_3_), 4.60–4.65 (*m*, 1H, Asp αH), 5.56 (*d*, *J* = 4.8 Hz, 1H, Asp NH).

Compound **2** was synthesized according to a slightly modified literature procedure (Wang *et al.*, 1977 ▸). Compound **1** (7.05 g, 28.5 mmol) was dissolved in MeOH (20 mL) and 0.7 *M* aqueous Cs_2_CO_3_ solution (20 ml) was added. The mixture was evaporated to dryness and the residue was re-evaporated three times with EtOH. A mixture of the white solid cesium salt and phenacyl bromide (5.68 g, 28.5 mmol) in DMF (30 mL) was stirred for 15min, and the precipitated cesium bromide removed. The solution was evaporated to give the residue, which was diluted with ethyl acetate, washed with water, sat. aqueous NaHCO_3_, and dried over Na_2_SO_4_. The drying agent was filtered off and the filtrate evaporated under reduced pressure. Crystallization of the product from a mixture of ethyl acetate and hexane afforded colourless crystals. Yield 5.36 g (14.7 mmol, 51.5%). ^1^H NMR (400 MHz, CDCl_3_): δ 1.46 (*s*, 9H, Boc *t*-but­yl), 2.93–2.99 (*m*, 1H, Asp βH), 3.07–3.12 (*m*, 1H, Asp βH), 3.74 (*s*, 3H, Asp OCH_3_), 4.77–4.82 (*m*, 1H, Asp αH), 5.35–5.48 (*m*, 2H, Pac CH_2_), 5.59 (*d*, *J* = 8.8 Hz, Asp NH), 7.47–7.52 (*m*, 2H, Pac phen­yl), 7.60–7.64 (*m*, 1H, Pac phen­yl), 7.89–7.91 (*m*, 2H, Pac phen­yl).

Compound **3**: Compound **2** (0.67 g, 2.72 mmol) was treated with 4.0 *M* HCl in dioxane for 60 min. The excess of HCl and solvent were evaporated and the residue was re-evaporated three times with MeOH, which was used for the next reaction without purification.

Compound **4**: A solution of compound **1** (1.01 g, 2.72 mmol), compound **3** (2.72 mmol), 2-(1*H*-benzotriazol-1-yl)-1,1,3,3-tetra­methyl­uronium hexa­fluorido­phosphate (HBTU; 1.24 g, 3.26 mmol), 1,2,3-benzotriazol-1-ol monohydrate (HOBt; 0.44 g, 3.26 mmol) and ^*i*^Pr_2_NEt (1.11 ml, 6.52 mmol) in DMF was stirred at room temperature for 20 h. The solution was then evaporated, diluted with ethyl acetate, washed with sat. aqueous KHSO_4_ and sat. aqueous NaHCO_3_, and dried over Na_2_SO_4_. After evaporation of the solvent, the residue was purified by column chromatography on silica gel (50% EtOAc in *n*-hexa­ne). Crystallization of the product from a mixture of ethyl acetate and hexane (*v*:*v* = 1:1) afforded colourless crystals. Yield 0.70 g (1.42 mmol, 52.4%). ^1^H NMR (400 MHz, CDCl_3_): δ 1.45 (*s*, 9H, Boc *t*-but­yl), 2.71–3.03 (*m*, 2H, Asp βH), 2.95–3.13 (*m*, 2H, Asp βH), 3.70 (*s*, 3H, Asp OCH_3_), 3.75 (*s*, 3H, Asp OCH_3_), 4.58–4.60 (*m*, 1H, Asp αH), 5.03–5.08 (*m*, 1H, Asp αH), 5.35–5.47 (*m*, 2H, Pac CH_2_), 5.71 (*d*, *J* = 8.4 Hz, 1H, Asp NH), 7.47–7.51 (*m*, 2H, Pac phen­yl), 7.58 (*d*, *J* = 8.4 Hz, 1H, Asp NH), 7.60–7.64 (*m*, 2H, Pac phen­yl), 7.88–7.90 (*m*, 2H, Pac phen­yl).

Compound **5**: Compound **4** (101.2 mg, 0.212 mmol) was treated with 4.0 *M* HCl in dioxane for 60 min. The excess of HCl and solvent were evaporated and the residue was re-evaporated three times with MeOH, which was used for the next reaction without purification.

Compound **6**: A solution of compound **1** (57.7 mg, 0.233 mmol), compound **5** (0.212 mmol), 1-ethyl-3-(3-di­methyl­amino­prop­yl)carbodi­imide hydro­chloride (WSCHCl; 55.2 mg, 0.288 mmol), HOBt (37.1 mg, 0.275 mmol) and Et_3_N (71 µl, 0.509 mmol) in DMF was stirred at room temperature for 20 h. The solution was then evaporated, diluted with ethyl acetate, washed with sat. aqueous KHSO_4_ and sat. aqueous NaHCO_3_, and dried over Na_2_SO_4_. After evaporation of the solvent, the residue was purified by column chromatography on silica gel (66% EtOAc in *n*–hexa­ne). Crystallization of the product from a mixture of ethyl acetate and hexane (*v*:*v* = 1:1) afforded colourless crystals. Yield 77 mg (0.127 mmol, 59.9%). ^1^H NMR (400 MHz, CDCl_3_): δ 1.45 (*s*, 9H, Boc *t*-but­yl), 2.72–2.82 (*m*, 2H, Asp βH), 2.96–3.04 (*m*, 4H, Asp βH), 3.69 (*s*, 3H, Asp OCH_3_), 3.71 (*s*, 3H, Asp OCH_3_), 3.74 (*s*, 3H, Asp OCH_3_), 4.52–4.56 (*m*,1H, Asp αH), 4.83–4.88 (*m*, 1H, Asp αH), 5.03–5.08 (*m*, 1H, Asp aH), 5.33–5.46 (*m*, 2H, Pac CH_2_), 5.65 (*d*, *J* = 8.4Hz, 1H, Asp NH), 7.47–7.51 (*m*, 1H, Pac phen­yl), 7.60–7.64 (*m*, 3H, Pac phenyl, Asp NH), 7.87–7.89 (*m*, 2H, Pac phen­yl). Single crystals suitable for X-ray diffraction were obtained by slow evaporation from a solution of acetone/water (19:1 *v*/*v*) mixture.

## Refinement   

Crystal data, data collection and structure refinement details are summarized in Table 3 ▸. The Boc protecting group at the *N*-terminus of the peptide is disordered. The final occupancy ratio is 0.504 (5):0.496 (5). The C atoms of the disordered *tert*-butyl groups were refined with SIMU restraints and the C5—O5*A* and C5—O5*B* bonds were treated with DFIX restraints of 1.21 (1) Å. The N-bound H atoms were refined freely, while the other H atoms were placed in geometrically idealized positions (C—H = 0.95–1.00 Å) and refined as riding on their parent atoms, with *U*_iso_(H) = 1.2*U*_eq_(C) (or 1.5*U*_eq_(C) for the methyl groups). The absolute configuration was known for the synthesized material.

## Supplementary Material

Crystal structure: contains datablock(s) I. DOI: 10.1107/S2056989019004596/is5511sup1.cif

Structure factors: contains datablock(s) I. DOI: 10.1107/S2056989019004596/is5511Isup2.hkl

Supporting information file. DOI: 10.1107/S2056989019004596/is5511Isup3.cdx

CCDC reference: 1907978

Additional supporting information:  crystallographic information; 3D view; checkCIF report

## Figures and Tables

**Table 1:** Selected torsion angles (°)

Name	Atoms	Torsion angle
ω_0*A*_	O4*A*—C5—N1—C11	169.2 (3)
ω_0*B*_	O4*B*—C5—N1—C11	−167.9 (2)
φ_1_	C5—N1—C11—C12	−122.49 (17)
ψ_1_	N1—C11—C12—N2	86.49 (17)
ω_1_	C11—C12—N2—C21	−168.62 (12)
φ_2_	C12—N2—C21—C22	−116.98 (16)
ψ_2_	N2—C21—C22—N3	112.58 (16)
ω_2_	C21—C22—N3—C31	−173.95 (13)
φ_3_	C22—N3—C31—C32	−84.60 (18)
χ_1_	N1—C11—C13—C14	172.69 (15)
χ_2_	N2—C21—C23—C24	−66.56 (19)
χ_3_	N3—C31—C33—C34	−57.72 (17)

**Table 2:** Hydrogen-bond geometry (Å, °)

*D*—H⋯*A*	*D*—H	H⋯*A*	*D*⋯*A*	*D*—H⋯*A*
N1—H1⋯O5*A* ^i^	0.80 (3)	2.13 (3)	2.867 (11)	154 (2)
N1—H1⋯O5*B* ^i^	0.80 (3)	2.04 (3)	2.800 (10)	158 (2)
N2—H2⋯O12^ii^	0.89 (3)	2.22 (3)	3.066 (2)	159.5 (18)
N3—H3⋯O22^i^	0.85 (2)	2.16 (2)	2.995 (2)	170.1 (19)
C2*A*—H2*A*1⋯O4*A* ^ii^	0.98	2.56	3.290 (12)	131
C13—H13*B*⋯O5*A* ^i^	0.99	2.48	3.300 (18)	140
C13—H13*B*⋯O5*B* ^i^	0.99	2.55	3.345 (17)	137
C15—H15*B*⋯O34^iii^	0.98	2.36	3.155 (2)	138
C25—H25*B*⋯O32^iv^	0.98	2.53	3.419 (2)	151
C31—H31⋯O35^ii^	1.00	2.34	3.319 (2)	164
C33—H33*A*⋯O41^i^	0.99	2.42	3.385 (2)	165
C41—H41*A*⋯O42^ii^	0.99	2.35	3.319 (2)	165
C46—H46⋯O34^v^	0.95	2.59	3.543 (3)	176

Symmetry codes: (i) 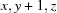; (ii) 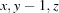; (iii) 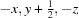; (iv) 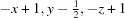; (v) 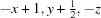.

**Table 3:** Experimental details

Crystal data	
Chemical formula	C_28_H_37_N_3_O_13_
*M*_r_	623.60
Crystal system, space group	Monoclinic, *P*2_1_
Temperature (K)	93
*a*, *b*, *c* (Å)	17.7734 (2), 4.97864 (4), 18.7681 (2)
β (°)	114.2255 (14)
*V* (Å^3^)	1514.49 (3)
*Z*	2
Radiation type	Cu *K*α
μ (mm^−1^)	0.93
Crystal size (mm)	0.41 × 0.14 × 0.04

Data collection	
Diffractometer	Rigaku Oxford Diffraction XtaLAB Pro: Kappa single
Absorption correction	Multi-scan (*CrysAlis PRO*; Rigaku OD, 2015 ▸)
*T*_min_, *T*_max_	0.754, 1.000
No. of measured, independent and observed [*I* > 2σ(*I*)] reflections	7024, 3952, 3832
*R*_int_	0.018
(sin θ/λ)_max_ (Å^−1^)	0.625

Refinement	
*R*[*F* ^2^ > 2σ(*F* ^2^)], *wR*(*F* ^2^), *S*	0.026, 0.072, 0.77
No. of reflections	3952
No. of parameters	470
No. of restraints	21
H-atom treatment	H atoms treated by a mixture of independent and constrained refinement
Δρ_max_, Δρ_min_ (e Å^−3^)	0.17, −0.18
Absolute structure	Flack *x* determined using 629 quotients [(*I* ^+^)−(*I* ^−^)]/[(*I* ^+^)+(*I* ^−^)] (Parsons *et al.*, 2013 ▸)
Absolute structure parameter	0.06 (12)

Computer programs: *CrysAlis PRO* (Rigaku OD, 2015 ▸), *SHELXS* (Sheldrick, 2008 ▸), *SHELXL2016/6* (Sheldrick, 2015 ▸), *PLATON* (Spek, 2009 ▸) and *publCIF* (Westrip, 2010 ▸).

## supplementary crystallographic information

### Crystal data

**Table:**

C_28_H_37_N_3_O_13_	*F*(000) = 660
*M**_r_* = 623.60	*D*_x_ = 1.367 Mg m^−^^3^
Monoclinic, *P*2_1_	Cu *K*α radiation, λ = 1.54184 Å
*a* = 17.7734 (2) Å	Cell parameters from 5605 reflections
*b* = 4.97864 (4) Å	θ = 4.8–74.0°
*c* = 18.7681 (2) Å	µ = 0.93 mm^−^^1^
β = 114.2255 (14)°	*T* = 93 K
*V* = 1514.49 (3) Å^3^	Plate, colorless
*Z* = 2	0.41 × 0.14 × 0.04 mm

### Data collection

**Table:**

Rigaku Oxford Diffraction XtaLAB Pro: Kappa single diffractometer	3952 independent reflections
Radiation source: fine-focus sealed X-ray tube	3832 reflections with *I* > 2σ(*I*)
Detector resolution: 5.811 pixels mm^-1^	*R*_int_ = 0.018
ω scans	θ_max_ = 74.4°, θ_min_ = 2.6°
Absorption correction: multi-scan (CrysAlisPro; Rigaku OD, 2015)	*h* = −21→21
*T*_min_ = 0.754, *T*_max_ = 1.000	*k* = −6→3
7024 measured reflections	*l* = −23→23

### Refinement

**Table:**

Refinement on *F*^2^	Hydrogen site location: mixed
Least-squares matrix: full	H atoms treated by a mixture of independent and constrained refinement
*R*[*F*^2^ > 2σ(*F*^2^)] = 0.026	*w* = 1/[σ^2^(*F*_o_^2^) + (0.0741*P*)^2^ + 0.2745*P*] where *P* = (*F*_o_^2^ + 2*F*_c_^2^)/3
*wR*(*F*^2^) = 0.072	(Δ/σ)_max_ < 0.001
*S* = 0.77	Δρ_max_ = 0.17 e Å^−^^3^
3952 reflections	Δρ_min_ = −0.18 e Å^−^^3^
470 parameters	Absolute structure: Flack *x* determined using 629 quotients [(*I*^+^)-(*I*^-^)]/[(*I*^+^)+(*I*^-^)] (Parsons *et al.*, 2013)
21 restraints	Absolute structure parameter: 0.06 (12)

### Special details

**Table:**

Geometry. All esds (except the esd in the dihedral angle between two l.s. planes) are estimated using the full covariance matrix. The cell esds are taken into account individually in the estimation of esds in distances, angles and torsion angles; correlations between esds in cell parameters are only used when they are defined by crystal symmetry. An approximate (isotropic) treatment of cell esds is used for estimating esds involving l.s. planes.

### Fractional atomic coordinates and isotropic or equivalent isotropic displacement parameters (Å^2^)

**Table:**

	*x*	*y*	*z*	*U*_iso_*/*U*_eq_	Occ. (<1)
C1A	0.2144 (3)	0.2721 (9)	0.6848 (2)	0.0345 (9)	0.504 (5)
H1A1	0.267469	0.355241	0.693593	0.052*	0.504 (5)
H1A2	0.220969	0.157562	0.729453	0.052*	0.504 (5)
H1A3	0.173686	0.412611	0.679050	0.052*	0.504 (5)
C2A	0.2520 (6)	−0.0962 (18)	0.6151 (5)	0.0266 (13)	0.504 (5)
H2A1	0.231399	−0.213264	0.569073	0.040*	0.504 (5)
H2A2	0.267358	−0.205521	0.662432	0.040*	0.504 (5)
H2A3	0.300386	0.002635	0.616886	0.040*	0.504 (5)
C3A	0.1022 (5)	−0.0283 (18)	0.5951 (5)	0.0263 (14)	0.504 (5)
H3A1	0.060178	0.111495	0.584750	0.039*	0.504 (5)
H3A2	0.106814	−0.132737	0.640966	0.039*	0.504 (5)
H3A3	0.086230	−0.147514	0.549665	0.039*	0.504 (5)
C4A	0.1848 (4)	0.102 (2)	0.6103 (5)	0.0217 (15)	0.504 (5)
O4A	0.1746 (3)	0.3070 (16)	0.5505 (4)	0.0261 (11)	0.504 (5)
O5A	0.1213 (9)	0.008 (2)	0.4475 (13)	0.024 (2)	0.504 (5)
C1B	0.1534 (3)	0.2751 (9)	0.6701 (2)	0.0328 (9)	0.496 (5)
H1B1	0.199436	0.402629	0.686695	0.049*	0.496 (5)
H1B2	0.159098	0.158167	0.714055	0.049*	0.496 (5)
H1B3	0.101223	0.373908	0.652870	0.049*	0.496 (5)
C2B	0.2381 (6)	−0.0274 (18)	0.6260 (6)	0.0288 (14)	0.496 (5)
H2B1	0.237862	−0.137167	0.582533	0.043*	0.496 (5)
H2B2	0.249930	−0.141679	0.671843	0.043*	0.496 (5)
H2B3	0.280682	0.111625	0.638512	0.043*	0.496 (5)
C3B	0.0818 (5)	−0.0926 (18)	0.5758 (5)	0.0254 (13)	0.496 (5)
H3B1	0.029814	0.006775	0.551414	0.038*	0.496 (5)
H3B2	0.082257	−0.192873	0.620932	0.038*	0.496 (5)
H3B3	0.086929	−0.218099	0.537838	0.038*	0.496 (5)
C4B	0.1541 (5)	0.105 (3)	0.6028 (6)	0.0212 (15)	0.496 (5)
O4B	0.1433 (3)	0.3082 (16)	0.5416 (4)	0.0220 (10)	0.496 (5)
O5B	0.1396 (9)	0.001 (2)	0.4505 (12)	0.024 (2)	0.496 (5)
C5	0.14313 (11)	0.2319 (4)	0.47256 (9)	0.0202 (4)	
N1	0.14230 (10)	0.4450 (3)	0.42909 (8)	0.0207 (3)	
H1	0.1496 (14)	0.593 (6)	0.4473 (13)	0.025*	
C11	0.12630 (9)	0.4285 (4)	0.34665 (8)	0.0159 (3)	
H11	0.119307	0.236491	0.329481	0.019*	
C12	0.20103 (9)	0.5508 (4)	0.33717 (8)	0.0152 (3)	
O12	0.20389 (7)	0.7897 (3)	0.32340 (7)	0.0207 (3)	
C13	0.04814 (9)	0.5864 (4)	0.29904 (9)	0.0198 (4)	
H13A	0.000362	0.496514	0.303103	0.024*	
H13B	0.052796	0.768642	0.321538	0.024*	
C14	0.03251 (9)	0.6103 (4)	0.21412 (9)	0.0191 (3)	
O14	0.05911 (8)	0.4612 (3)	0.17994 (7)	0.0285 (3)	
C15	−0.03784 (11)	0.8735 (5)	0.09979 (10)	0.0293 (4)	
H15A	−0.009289	1.036228	0.094924	0.044*	
H15B	−0.097570	0.898998	0.072156	0.044*	
H15C	−0.020654	0.721049	0.076939	0.044*	
O15	−0.01716 (7)	0.8205 (3)	0.18149 (7)	0.0269 (3)	
N2	0.26296 (8)	0.3747 (3)	0.34922 (8)	0.0159 (3)	
H2	0.2565 (12)	0.199 (6)	0.3536 (11)	0.019*	
C21	0.34413 (9)	0.4635 (4)	0.35689 (8)	0.0156 (3)	
H21	0.344606	0.663962	0.353802	0.019*	
C22	0.36114 (9)	0.3452 (4)	0.28995 (9)	0.0160 (3)	
O22	0.37002 (7)	0.1028 (3)	0.28428 (6)	0.0206 (3)	
C23	0.41063 (9)	0.3731 (4)	0.43516 (8)	0.0185 (4)	
H23A	0.406738	0.176533	0.440900	0.022*	
H23B	0.465823	0.413628	0.436741	0.022*	
C24	0.40034 (9)	0.5149 (4)	0.50166 (9)	0.0187 (3)	
O24	0.36527 (8)	0.7236 (3)	0.49676 (7)	0.0276 (3)	
O25	0.43715 (7)	0.3764 (3)	0.56861 (6)	0.0243 (3)	
C25	0.43003 (11)	0.4982 (5)	0.63560 (9)	0.0289 (4)	
H25A	0.450425	0.683342	0.641563	0.043*	
H25B	0.462802	0.395351	0.682707	0.043*	
H25C	0.372054	0.498328	0.627883	0.043*	
N3	0.36442 (8)	0.5253 (3)	0.23773 (8)	0.0165 (3)	
H3	0.3610 (12)	0.692 (5)	0.2455 (12)	0.020*	
C31	0.37213 (9)	0.4317 (4)	0.16774 (8)	0.0158 (3)	
H31	0.340229	0.260920	0.150068	0.019*	
C32	0.46177 (10)	0.3789 (4)	0.18265 (8)	0.0173 (3)	
O32	0.52056 (7)	0.5030 (3)	0.22640 (6)	0.0235 (3)	
C33	0.33705 (10)	0.6411 (4)	0.10194 (9)	0.0179 (3)	
H33A	0.369066	0.810066	0.117588	0.021*	
H33B	0.340708	0.573229	0.053884	0.021*	
C34	0.24805 (10)	0.6920 (4)	0.08698 (9)	0.0183 (3)	
O34	0.19207 (7)	0.5396 (3)	0.05273 (7)	0.0262 (3)	
O35	0.24040 (7)	0.9215 (3)	0.11951 (7)	0.0227 (3)	
C35	0.15959 (11)	0.9750 (5)	0.11807 (12)	0.0319 (4)	
H35A	0.119401	0.996061	0.063845	0.048*	
H35B	0.161673	1.140387	0.147146	0.048*	
H35C	0.143100	0.824807	0.142250	0.048*	
O41	0.46534 (7)	0.1747 (3)	0.13693 (7)	0.0198 (3)	
C41	0.54548 (10)	0.1292 (4)	0.13817 (10)	0.0208 (4)	
H41A	0.546104	−0.044984	0.112960	0.025*	
H41B	0.587062	0.121432	0.192914	0.025*	
C42	0.56705 (10)	0.3543 (4)	0.09510 (9)	0.0178 (3)	
O42	0.51578 (7)	0.5211 (3)	0.05891 (6)	0.0216 (3)	
C43	0.65291 (10)	0.3610 (4)	0.09916 (8)	0.0182 (3)	
C44	0.67194 (10)	0.5559 (4)	0.05561 (9)	0.0205 (4)	
H44	0.630773	0.678584	0.024158	0.025*	
C45	0.71356 (10)	0.1801 (4)	0.14514 (9)	0.0217 (4)	
H45	0.700557	0.045572	0.174190	0.026*	
C46	0.75121 (11)	0.5701 (4)	0.05834 (9)	0.0231 (4)	
H46	0.764212	0.701551	0.028446	0.028*	
C47	0.79299 (11)	0.1973 (4)	0.14827 (10)	0.0246 (4)	
H47	0.834544	0.076250	0.180023	0.030*	
C48	0.81132 (10)	0.3916 (5)	0.10490 (10)	0.0241 (4)	
H48	0.865573	0.402778	0.107059	0.029*	

### Atomic displacement parameters (Å^2^)

**Table:**

	*U*^11^	*U*^22^	*U*^33^	*U*^12^	*U*^13^	*U*^23^
C1A	0.068 (2)	0.0176 (18)	0.0219 (14)	0.000 (2)	0.0225 (18)	0.0003 (14)
C2A	0.029 (3)	0.026 (4)	0.026 (2)	0.003 (3)	0.0132 (15)	0.003 (2)
C3A	0.029 (4)	0.026 (4)	0.030 (4)	0.001 (3)	0.018 (3)	0.008 (2)
C4A	0.038 (4)	0.015 (2)	0.016 (2)	−0.001 (5)	0.015 (4)	0.003 (2)
O4A	0.049 (3)	0.0146 (17)	0.0160 (18)	−0.003 (3)	0.014 (3)	−0.0002 (14)
O5A	0.036 (5)	0.016 (3)	0.025 (3)	−0.003 (2)	0.018 (4)	−0.003 (2)
C1B	0.067 (2)	0.0182 (18)	0.0208 (14)	−0.005 (2)	0.0254 (17)	0.0008 (14)
C2B	0.029 (3)	0.027 (4)	0.029 (3)	0.001 (3)	0.0113 (17)	0.007 (3)
C3B	0.028 (3)	0.021 (4)	0.030 (4)	−0.005 (2)	0.015 (3)	0.006 (2)
C4B	0.033 (4)	0.015 (2)	0.018 (2)	0.001 (4)	0.012 (3)	0.0045 (19)
O4B	0.040 (3)	0.0113 (16)	0.0166 (18)	−0.005 (3)	0.013 (2)	−0.0007 (14)
O5B	0.044 (6)	0.011 (2)	0.021 (2)	0.005 (2)	0.016 (4)	0.0022 (17)
C5	0.0293 (8)	0.0167 (9)	0.0176 (7)	0.0000 (8)	0.0127 (6)	−0.0003 (7)
N1	0.0357 (8)	0.0124 (8)	0.0170 (6)	−0.0036 (7)	0.0137 (6)	−0.0031 (6)
C11	0.0205 (7)	0.0154 (9)	0.0142 (6)	−0.0015 (7)	0.0094 (6)	−0.0012 (6)
C12	0.0184 (7)	0.0139 (9)	0.0131 (6)	0.0002 (7)	0.0063 (5)	0.0002 (6)
O12	0.0210 (6)	0.0148 (7)	0.0272 (6)	0.0008 (5)	0.0109 (5)	0.0030 (5)
C13	0.0197 (7)	0.0219 (9)	0.0205 (7)	0.0003 (7)	0.0110 (6)	−0.0001 (7)
C14	0.0153 (7)	0.0205 (9)	0.0196 (7)	−0.0035 (7)	0.0054 (6)	−0.0002 (7)
O14	0.0373 (7)	0.0275 (8)	0.0216 (5)	0.0052 (6)	0.0129 (5)	−0.0007 (6)
C15	0.0243 (8)	0.0346 (12)	0.0245 (8)	0.0022 (9)	0.0055 (6)	0.0090 (9)
O15	0.0246 (6)	0.0292 (8)	0.0251 (6)	0.0084 (6)	0.0083 (5)	0.0071 (6)
N2	0.0177 (6)	0.0113 (8)	0.0195 (6)	−0.0010 (6)	0.0083 (5)	0.0005 (6)
C21	0.0166 (7)	0.0149 (9)	0.0166 (7)	−0.0003 (6)	0.0083 (5)	−0.0003 (7)
C22	0.0142 (7)	0.0167 (10)	0.0167 (7)	−0.0006 (7)	0.0061 (6)	0.0001 (7)
O22	0.0303 (6)	0.0141 (7)	0.0193 (5)	0.0017 (5)	0.0119 (5)	0.0000 (5)
C23	0.0195 (7)	0.0188 (9)	0.0169 (7)	0.0024 (7)	0.0072 (6)	0.0002 (7)
C24	0.0181 (7)	0.0216 (9)	0.0152 (7)	−0.0017 (8)	0.0055 (5)	0.0003 (7)
O24	0.0384 (7)	0.0232 (8)	0.0205 (5)	0.0077 (6)	0.0114 (5)	−0.0015 (6)
O25	0.0315 (6)	0.0260 (7)	0.0149 (5)	0.0023 (6)	0.0091 (4)	0.0008 (5)
C25	0.0366 (9)	0.0360 (12)	0.0160 (7)	−0.0002 (10)	0.0127 (7)	−0.0015 (8)
N3	0.0232 (6)	0.0125 (8)	0.0173 (6)	−0.0011 (6)	0.0119 (5)	−0.0024 (6)
C31	0.0198 (7)	0.0136 (9)	0.0160 (6)	−0.0015 (6)	0.0095 (5)	−0.0019 (6)
C32	0.0233 (7)	0.0152 (9)	0.0159 (6)	0.0000 (7)	0.0106 (6)	0.0019 (7)
O32	0.0214 (5)	0.0270 (7)	0.0217 (5)	−0.0035 (6)	0.0085 (4)	−0.0061 (6)
C33	0.0210 (7)	0.0172 (9)	0.0172 (7)	−0.0015 (7)	0.0096 (6)	0.0004 (7)
C34	0.0227 (8)	0.0181 (9)	0.0143 (6)	−0.0004 (7)	0.0078 (6)	0.0029 (7)
O34	0.0236 (6)	0.0286 (8)	0.0235 (5)	−0.0072 (6)	0.0066 (5)	−0.0050 (6)
O35	0.0214 (6)	0.0189 (7)	0.0319 (6)	−0.0008 (5)	0.0151 (5)	−0.0021 (6)
C35	0.0280 (9)	0.0298 (12)	0.0474 (10)	0.0047 (9)	0.0249 (8)	0.0057 (10)
O41	0.0218 (5)	0.0169 (7)	0.0252 (5)	−0.0009 (5)	0.0142 (4)	−0.0043 (5)
C41	0.0224 (8)	0.0177 (10)	0.0279 (8)	0.0014 (7)	0.0159 (7)	0.0001 (7)
C42	0.0242 (7)	0.0147 (9)	0.0162 (6)	−0.0011 (7)	0.0102 (6)	−0.0049 (7)
O42	0.0246 (6)	0.0195 (7)	0.0221 (5)	0.0029 (5)	0.0107 (4)	0.0010 (5)
C43	0.0236 (8)	0.0164 (9)	0.0163 (6)	−0.0018 (7)	0.0098 (6)	−0.0030 (7)
C44	0.0251 (8)	0.0188 (10)	0.0181 (7)	−0.0009 (7)	0.0094 (6)	−0.0017 (7)
C45	0.0268 (8)	0.0195 (10)	0.0215 (7)	0.0007 (8)	0.0126 (6)	0.0009 (7)
C46	0.0298 (8)	0.0219 (10)	0.0216 (7)	−0.0063 (8)	0.0145 (7)	−0.0031 (7)
C47	0.0236 (8)	0.0240 (10)	0.0255 (8)	0.0014 (8)	0.0093 (6)	−0.0014 (8)
C48	0.0225 (8)	0.0275 (10)	0.0249 (8)	−0.0052 (8)	0.0123 (6)	−0.0063 (8)

### Geometric parameters (Å, º)

**Table:**

C1A—C4A	1.532 (11)	N2—H2	0.89 (3)
C1A—H1A1	0.9800	C21—C22	1.526 (2)
C1A—H1A2	0.9800	C21—C23	1.527 (2)
C1A—H1A3	0.9800	C21—H21	1.0000
C2A—C4A	1.521 (10)	C22—O22	1.227 (2)
C2A—H2A1	0.9800	C22—N3	1.347 (2)
C2A—H2A2	0.9800	C23—C24	1.509 (2)
C2A—H2A3	0.9800	C23—H23A	0.9900
C3A—C4A	1.521 (10)	C23—H23B	0.9900
C3A—H3A1	0.9800	C24—O24	1.196 (2)
C3A—H3A2	0.9800	C24—O25	1.345 (2)
C3A—H3A3	0.9800	O25—C25	1.447 (2)
C4A—O4A	1.473 (13)	C25—H25A	0.9800
O4A—C5	1.387 (7)	C25—H25B	0.9800
O5A—C5	1.212 (9)	C25—H25C	0.9800
C1B—C4B	1.527 (11)	N3—C31	1.4522 (19)
C1B—H1B1	0.9800	N3—H3	0.85 (3)
C1B—H1B2	0.9800	C31—C32	1.523 (2)
C1B—H1B3	0.9800	C31—C33	1.539 (2)
C2B—C4B	1.523 (10)	C31—H31	1.0000
C2B—H2B1	0.9800	C32—O32	1.202 (2)
C2B—H2B2	0.9800	C32—O41	1.348 (2)
C2B—H2B3	0.9800	C33—C34	1.510 (2)
C3B—C4B	1.529 (10)	C33—H33A	0.9900
C3B—H3B1	0.9800	C33—H33B	0.9900
C3B—H3B2	0.9800	C34—O34	1.206 (2)
C3B—H3B3	0.9800	C34—O35	1.328 (2)
C4B—O4B	1.484 (13)	O35—C35	1.450 (2)
O4B—C5	1.348 (7)	C35—H35A	0.9800
O5B—C5	1.216 (9)	C35—H35B	0.9800
C5—N1	1.335 (2)	C35—H35C	0.9800
N1—C11	1.4563 (18)	O41—C41	1.4329 (19)
N1—H1	0.80 (3)	C41—C42	1.520 (2)
C11—C13	1.526 (2)	C41—H41A	0.9900
C11—C12	1.536 (2)	C41—H41B	0.9900
C11—H11	1.0000	C42—O42	1.214 (2)
C12—O12	1.222 (2)	C42—C43	1.497 (2)
C12—N2	1.352 (2)	C43—C44	1.397 (2)
C13—C14	1.506 (2)	C43—C45	1.398 (2)
C13—H13A	0.9900	C44—C46	1.390 (2)
C13—H13B	0.9900	C44—H44	0.9500
C14—O14	1.198 (2)	C45—C47	1.391 (2)
C14—O15	1.344 (2)	C45—H45	0.9500
C15—O15	1.447 (2)	C46—C48	1.389 (3)
C15—H15A	0.9800	C46—H46	0.9500
C15—H15B	0.9800	C47—C48	1.386 (3)
C15—H15C	0.9800	C47—H47	0.9500
N2—C21	1.4586 (19)	C48—H48	0.9500
			
C4A—C1A—H1A1	109.5	C12—N2—C21	121.62 (16)
C4A—C1A—H1A2	109.5	C12—N2—H2	121.7 (14)
H1A1—C1A—H1A2	109.5	C21—N2—H2	116.7 (13)
C4A—C1A—H1A3	109.5	N2—C21—C22	108.90 (13)
H1A1—C1A—H1A3	109.5	N2—C21—C23	110.44 (13)
H1A2—C1A—H1A3	109.5	C22—C21—C23	109.98 (13)
C4A—C2A—H2A1	109.5	N2—C21—H21	109.2
C4A—C2A—H2A2	109.5	C22—C21—H21	109.2
H2A1—C2A—H2A2	109.5	C23—C21—H21	109.2
C4A—C2A—H2A3	109.5	O22—C22—N3	123.09 (15)
H2A1—C2A—H2A3	109.5	O22—C22—C21	121.80 (15)
H2A2—C2A—H2A3	109.5	N3—C22—C21	115.11 (16)
C4A—C3A—H3A1	109.5	C24—C23—C21	110.43 (14)
C4A—C3A—H3A2	109.5	C24—C23—H23A	109.6
H3A1—C3A—H3A2	109.5	C21—C23—H23A	109.6
C4A—C3A—H3A3	109.5	C24—C23—H23B	109.6
H3A1—C3A—H3A3	109.5	C21—C23—H23B	109.6
H3A2—C3A—H3A3	109.5	H23A—C23—H23B	108.1
O4A—C4A—C2A	110.1 (7)	O24—C24—O25	123.78 (15)
O4A—C4A—C3A	110.1 (6)	O24—C24—C23	125.39 (15)
C2A—C4A—C3A	114.2 (9)	O25—C24—C23	110.82 (16)
O4A—C4A—C1A	101.5 (8)	C24—O25—C25	114.48 (15)
C2A—C4A—C1A	109.8 (6)	O25—C25—H25A	109.5
C3A—C4A—C1A	110.5 (7)	O25—C25—H25B	109.5
C5—O4A—C4A	119.1 (7)	H25A—C25—H25B	109.5
C4B—C1B—H1B1	109.5	O25—C25—H25C	109.5
C4B—C1B—H1B2	109.5	H25A—C25—H25C	109.5
H1B1—C1B—H1B2	109.5	H25B—C25—H25C	109.5
C4B—C1B—H1B3	109.5	C22—N3—C31	119.51 (15)
H1B1—C1B—H1B3	109.5	C22—N3—H3	119.8 (14)
H1B2—C1B—H1B3	109.5	C31—N3—H3	120.7 (14)
C4B—C2B—H2B1	109.5	N3—C31—C32	111.65 (12)
C4B—C2B—H2B2	109.5	N3—C31—C33	110.54 (14)
H2B1—C2B—H2B2	109.5	C32—C31—C33	108.24 (12)
C4B—C2B—H2B3	109.5	N3—C31—H31	108.8
H2B1—C2B—H2B3	109.5	C32—C31—H31	108.8
H2B2—C2B—H2B3	109.5	C33—C31—H31	108.8
C4B—C3B—H3B1	109.5	O32—C32—O41	124.59 (15)
C4B—C3B—H3B2	109.5	O32—C32—C31	125.98 (16)
H3B1—C3B—H3B2	109.5	O41—C32—C31	109.40 (13)
C4B—C3B—H3B3	109.5	C34—C33—C31	108.13 (12)
H3B1—C3B—H3B3	109.5	C34—C33—H33A	110.1
H3B2—C3B—H3B3	109.5	C31—C33—H33A	110.1
O4B—C4B—C2B	108.5 (6)	C34—C33—H33B	110.1
O4B—C4B—C3B	111.0 (7)	C31—C33—H33B	110.1
C2B—C4B—C3B	114.2 (10)	H33A—C33—H33B	108.4
O4B—C4B—C1B	102.7 (8)	O34—C34—O35	124.64 (15)
C2B—C4B—C1B	110.1 (7)	O34—C34—C33	124.45 (17)
C3B—C4B—C1B	109.8 (6)	O35—C34—C33	110.85 (14)
C5—O4B—C4B	120.0 (7)	C34—O35—C35	115.91 (15)
O5A—C5—N1	125.0 (11)	O35—C35—H35A	109.5
O5B—C5—N1	124.0 (11)	O35—C35—H35B	109.5
O5B—C5—O4B	124.9 (11)	H35A—C35—H35B	109.5
N1—C5—O4B	111.0 (4)	O35—C35—H35C	109.5
O5A—C5—O4A	125.8 (12)	H35A—C35—H35C	109.5
N1—C5—O4A	109.2 (4)	H35B—C35—H35C	109.5
C5—N1—C11	123.55 (15)	C32—O41—C41	114.52 (13)
C5—N1—H1	120.9 (16)	O41—C41—C42	109.92 (14)
C11—N1—H1	115.6 (16)	O41—C41—H41A	109.7
N1—C11—C13	109.09 (13)	C42—C41—H41A	109.7
N1—C11—C12	107.42 (12)	O41—C41—H41B	109.7
C13—C11—C12	110.52 (13)	C42—C41—H41B	109.7
N1—C11—H11	109.9	H41A—C41—H41B	108.2
C13—C11—H11	109.9	O42—C42—C43	121.93 (16)
C12—C11—H11	109.9	O42—C42—C41	120.44 (15)
O12—C12—N2	124.40 (16)	C43—C42—C41	117.63 (15)
O12—C12—C11	121.54 (15)	C44—C43—C45	119.89 (15)
N2—C12—C11	113.98 (15)	C44—C43—C42	118.18 (15)
C14—C13—C11	112.88 (13)	C45—C43—C42	121.93 (16)
C14—C13—H13A	109.0	C46—C44—C43	119.93 (16)
C11—C13—H13A	109.0	C46—C44—H44	120.0
C14—C13—H13B	109.0	C43—C44—H44	120.0
C11—C13—H13B	109.0	C47—C45—C43	119.89 (17)
H13A—C13—H13B	107.8	C47—C45—H45	120.1
O14—C14—O15	124.62 (15)	C43—C45—H45	120.1
O14—C14—C13	125.16 (17)	C48—C46—C44	119.78 (17)
O15—C14—C13	110.21 (15)	C48—C46—H46	120.1
O15—C15—H15A	109.5	C44—C46—H46	120.1
O15—C15—H15B	109.5	C48—C47—C45	119.80 (17)
H15A—C15—H15B	109.5	C48—C47—H47	120.1
O15—C15—H15C	109.5	C45—C47—H47	120.1
H15A—C15—H15C	109.5	C47—C48—C46	120.71 (16)
H15B—C15—H15C	109.5	C47—C48—H48	119.6
C14—O15—C15	117.30 (14)	C46—C48—H48	119.6
			
C2A—C4A—O4A—C5	67.6 (7)	C21—C23—C24—O24	−22.5 (2)
C3A—C4A—O4A—C5	−59.1 (8)	C21—C23—C24—O25	158.28 (14)
C1A—C4A—O4A—C5	−176.2 (4)	O24—C24—O25—C25	1.0 (2)
C2B—C4B—O4B—C5	61.7 (8)	C23—C24—O25—C25	−179.82 (14)
C3B—C4B—O4B—C5	−64.5 (8)	O22—C22—N3—C31	5.2 (2)
C1B—C4B—O4B—C5	178.2 (4)	C21—C22—N3—C31	−173.95 (13)
C4B—O4B—C5—O5B	11.0 (10)	C22—N3—C31—C32	−84.60 (18)
C4B—O4B—C5—N1	−172.8 (4)	C22—N3—C31—C33	154.84 (14)
C4A—O4A—C5—O5A	1.2 (10)	N3—C31—C32—O32	−34.3 (2)
C4A—O4A—C5—N1	−177.4 (4)	C33—C31—C32—O32	87.64 (19)
O5A—C5—N1—C11	−9.4 (9)	N3—C31—C32—O41	147.76 (14)
O5B—C5—N1—C11	8.3 (9)	C33—C31—C32—O41	−90.35 (16)
O4B—C5—N1—C11	−167.9 (2)	N3—C31—C33—C34	−57.72 (17)
O4A—C5—N1—C11	169.2 (3)	C32—C31—C33—C34	179.70 (14)
C5—N1—C11—C13	117.68 (19)	C31—C33—C34—O34	−75.17 (19)
C5—N1—C11—C12	−122.49 (17)	C31—C33—C34—O35	102.17 (16)
N1—C11—C12—O12	−90.42 (18)	O34—C34—O35—C35	5.2 (2)
C13—C11—C12—O12	28.5 (2)	C33—C34—O35—C35	−172.12 (14)
N1—C11—C12—N2	86.49 (17)	O32—C32—O41—C41	−5.8 (2)
C13—C11—C12—N2	−154.60 (13)	C31—C32—O41—C41	172.21 (13)
N1—C11—C13—C14	172.69 (15)	C32—O41—C41—C42	−71.55 (17)
C12—C11—C13—C14	54.80 (19)	O41—C41—C42—O42	−7.9 (2)
C11—C13—C14—O14	22.9 (3)	O41—C41—C42—C43	172.15 (13)
C11—C13—C14—O15	−158.40 (14)	O42—C42—C43—C44	−4.2 (2)
O14—C14—O15—C15	−1.8 (3)	C41—C42—C43—C44	175.78 (15)
C13—C14—O15—C15	179.47 (15)	O42—C42—C43—C45	175.55 (16)
O12—C12—N2—C21	8.2 (2)	C41—C42—C43—C45	−4.5 (2)
C11—C12—N2—C21	−168.62 (12)	C45—C43—C44—C46	−0.3 (3)
C12—N2—C21—C22	−116.98 (16)	C42—C43—C44—C46	179.46 (15)
C12—N2—C21—C23	122.15 (16)	C44—C43—C45—C47	1.0 (3)
N2—C21—C22—O22	−66.5 (2)	C42—C43—C45—C47	−178.75 (16)
C23—C21—C22—O22	54.6 (2)	C43—C44—C46—C48	−0.5 (3)
N2—C21—C22—N3	112.58 (16)	C43—C45—C47—C48	−0.9 (3)
C23—C21—C22—N3	−126.27 (15)	C45—C47—C48—C46	0.1 (3)
N2—C21—C23—C24	−66.56 (19)	C44—C46—C48—C47	0.6 (3)
C22—C21—C23—C24	173.22 (14)		

### Hydrogen-bond geometry (Å, º)

**Table:**

*D*—H···*A*	*D*—H	H···*A*	*D*···*A*	*D*—H···*A*
N1—H1···O5*A*^i^	0.80 (3)	2.13 (3)	2.867 (11)	154 (2)
N1—H1···O5*B*^i^	0.80 (3)	2.04 (3)	2.800 (10)	158 (2)
N2—H2···O12^ii^	0.89 (3)	2.22 (3)	3.066 (2)	159.5 (18)
N3—H3···O22^i^	0.85 (2)	2.16 (2)	2.995 (2)	170.1 (19)
C2*A*—H2*A*1···O4*A*^ii^	0.98	2.56	3.290 (12)	131
C13—H13*B*···O5*A*^i^	0.99	2.48	3.300 (18)	140
C13—H13*B*···O5*B*^i^	0.99	2.55	3.345 (17)	137
C15—H15*B*···O34^iii^	0.98	2.36	3.155 (2)	138
C25—H25*B*···O32^iv^	0.98	2.53	3.419 (2)	151
C31—H31···O35^ii^	1.00	2.34	3.319 (2)	164
C33—H33*A*···O41^i^	0.99	2.42	3.385 (2)	165
C41—H41*A*···O42^ii^	0.99	2.35	3.319 (2)	165
C46—H46···O34^v^	0.95	2.59	3.543 (3)	176

Symmetry codes: (i) *x*, *y*+1, *z*; (ii) *x*, *y*−1, *z*; (iii) −*x*, *y*+1/2, −*z*; (iv) −*x*+1, *y*−1/2, −*z*+1; (v) −*x*+1, *y*+1/2, −*z*.
